# The upstream regulation of the root cell wall when *Arabidopsis thaliana* in response to toxic metal ions focusing on Al

**DOI:** 10.1080/15592324.2023.2178085

**Published:** 2023-02-13

**Authors:** Ye Tao, Qi Wu, Jing Huang, Ren Fang Shen, Xiao Fang Zhu

**Affiliations:** State Key Laboratory of Soil and Sustainable Agriculture, Institute of Soil Science, Chinese Academy of Sciences, Nanjing, Jiangsu, China

**Keywords:** Aluminum (Al), cell wall, toxic metal ions, regulation, *Arabidopsis thaliana*

## Abstract

In acid soil, aluminum (Al) toxicity is one of the main factors limiting agricultural output. As is known to all, the cell wall is the first line of defense against metals that serves as a significant target of Al toxicity and also is crucial for Al detoxification. However, nothing is known about how this process is transcriptionally regulated. Here, we describe recent findings to understand the role of two kinds of transcription factors in regulating the cell wall composition and modification in response to Al stress in *Arabidopsis thaliana. ANAC017* encodes a NAM, ATAF1/2, and cup-shaped cotyledon 2 (NAC) transcription factor, loss function of *ANAC017* enhanced Al tolerance with the decreased Al content and xyloglucan content in the cell wall. Next, we characterized one xyloglucan endotransglucosylase/hydrolase (XTH), XTH31, which is previously reported to participate in Al stress, acted downstream of ANAC017 to regulate Al tolerance in *Arabidopsis*. In addition, we also identified MYB103, an R2R3-type transcription factor. *MYB103* disruption caused Al sensitivity, and *myb103* mutants’ xyloglucan had a high *O*-acetylation level. Additionally, it was discovered that TRICHOME BIREFRINGENCE-LIKE27 (TBL27), which is in charge of xyloglucan’s *O*-acetylation, functions downstream of MYB103 through the direct binding of the MYB103 to the promoter of the TBL27 to influence Arabidopsis’s sensitivity to Al. In summary, our research showed that two distinct molecular modules modulate Arabidopsis cell wall composition and modification to positively influence Al resistance.

Al is the most common metal in the crust of the planet, mainly in the two main forms of aluminosilicate and bauxite. However, with the increasing amount usage of chemical fertilizers in agricultural production and the strenuous leaching effect of acid rain in industrial activities, the soil pH value falls below 5 and then Al ion is easily released from the clay minerals which is named as soil acidification and causes the Al toxicity which seriously affect the plant growth.^1^ Therefore, any strategies that enhance the Al tolerance in crops could increase the crop yields and solve the issue of food shortage all around the world.

The cell wall is made up of pectin, hemicellulose, and cellulose. It is said that Al is combined with phosphate or carboxyl groups in plant cell walls, causing cell death or restriction and cell wall enlargement.^2^ Cell wall polysaccharides play a special role in rice root tip rejection of Al during Al toxicity.^3^ Despite the fact that cell walls play a crucial in role in Al stress response, little is known about the molecular mechanism that controls cell wall change during Al stress adaptation. The understanding of how the plant cell wall reacts to this Al stress has advanced significantly over the last 10 years, according to the researches.

Reports on the molecular factors governing cell wall modification under Al excessive condition started in the past 10 years. The hemicellulose fraction, particularly xyloglucan, which is a significant component of the primary cell wall hemicellulose of Arabidopsis and is easier to bind Al.^2^ The disruption of either XYLOGLUCAN ENDOTRANSGLUCOSYLASE HYDROLASE 17 (XTH17) or XTH31 reduces the ability of the root cell wall to bind to Al, which results in less xyloglucan content ^2,4^. The regulatory mechanisms of these cell wall-related genes, which are crucial for the xyloglucan component in the process of detoxifying excessive Al, are, however, poorly understood.

*LEUNIG HOMOLOG* (*LUH*) is a member of Groucho-like family transcriptional co-repressors, and the *luh* Arabidopsis mutants are less sensitive to Al-induced inhibition of root growth. Increased levels of methylated pectin and altered gene expression in the cell walls of *luh* roots are both associated with this trait. *PECTIN METHYLESTERASE46* (*PME46*) was shown to be one of the *LUH*-repressed genes that lessen cell wall-bound Al, thereby reducing Al-induced inhibition of root development by reducing PME enzyme activity. As with *luh* mutants, *seuss-like 2* (*slk2*) mutants responded to Al similarly, indicating that the LUH-SLK2 complex inhibits the expression of PME46.^5^ Additionally, the NAC-type transcription factor VuNAR1 of rice bean (*Vigna umbellata*) was described to be responsive to Al stress. Al selectively increased the expression of *VuNAR1*, a nuclear-localized transcriptional activator, in roots but not in shoots. On the basis of transcriptome studies, it is found that VuNAR1 uniquely regulates the expression of genes in *Arabidopsis thaliana*, which are related to protein phosphorylation and cell wall modification. A transient expression test showed that VuNAR1 directly activated the transcription of the *cell wall-associated receptor kinase 1* (*WAK1*). In addition, yeast one-hybrid experiments found that a new VuNAR1-specific binding motif in the *WAK1* promoter was found by yeast one-hybrid experiments, which suggested that *VuNAR1* controls Al resistance via controlling the metabolism of cell wall pectin by directly binding to the *WAK1* promoter and inducing the expression of *WAK1*.^6^ *RESISTANCE TO ALUMINUM 1* (*RAL1*) was cloned through the characterization of an Al-resistant rice mutant, which encodes an enzyme that may take part in lignin production, 4-coumarate: coenzyme A ligase 4CL4. Mutation of *RAL1*/*4CL4* results in reduced lignin production and then increased the accumulation of 4-coumaric acid (PA) and ferulic acid as substrates. The increase in Al resistance of *ral1*/*4cl4* mutants is not caused by altered lignin buildup. The accumulation of PA and FA induced by Al can actively participate in the resistance of rice to Al stress, mainly by modifying the cell wall and thereby reducing Al binding to the cell wall. This demonstrates that the increased resistance of *ral1*/*4cl4* mutants to Al was due to the decreased ability of Al to bind to hemicellulose by increasing PA and FA accumulation.^7^ Here, after extensive screening, we described ANAC017, a transcription factor that responds to Al stress in *Arabidopsis thaliana*. The expression of ANAC017 was constitutive in the roots and shoots, and the ANAC017 was localized in the nucleus. Al tolerance increased when ANAC017 function was deficient by lowering the level of hemicellulose and xyloglucan, pointing to a possible cross-link between ANAC017 and XTH. Furthermore, based on evidences from biochemistry and genetics, a novel ANAC017-XTH31 module was identified to regulate Al tolerance by affecting the cell wall composition, especially for xyloglucan.^8^

In addition to xyloglucan content itself, post-translational modification is also recognized as one important regulation response to Al stress. For example, the absence of *TRICHOME BIREFRINGENCE-LIKE27* (*TBL27*) increases the ability of the cell wall of Arabidopsis root to bind to Al by decreasing the degree of xyloglucan’s *O-*acetylation. Additionally, it has been suggested that glucuronoxylan, another significant hemicellulose component, controls the ability of the cell wall to bind Al by altering its α-D-glucuronic acid side chains^9,10^. Despite these breakthroughs, one of the most interesting questions remained: How the different cell wall processes especially for hemicellulose modification are coordinately regulated?

A large-scale mutants’ screening was carried out using the differentially expressed genes, and ultimately, the *myb103* mutant was identified as being more sensitive to Al stress, indicating greater root growth inhibition and increased root Al content. The answers to this question evolved from a public RNA-seq database about the analysis of transcriptional expression patterns under Al stress [http://ipf.sustech.edu.cn/pub/athrdb/;^11^]. MYB103, a previously described nuclear-localized transcription factor, is a member of the gene family that codes for transcription factors of the R2R3 type. Al exposure suppressed *MYB103*, and *MYB103* co-expressed with *TBL27* in roots, indicating that *MYB103* and *TBL27* may be related. More significantly, it was discovered that *myb103* mutants had higher Al accumulation of cell wall and hemicellulose in root, which was linked to variations in the *O*-acetylation level of xyloglucan rather than changes in the amount of xyloglucan itself. We used the approach of biochemistry, genetics and molecular biology comprehensively to reveal that MYB103-TBL27 regulatory module affects the sensitivity of Al by regulating the *O*-acetylation level of xyloglucan in the cell wall in order to better understand this MYB103-mediated regulatory.^11^

MYB103, an R2R3-type transcription factor, is previously reported as a nuclear-localized transcription factor.^12^ Al stress inhibited MYB103 and showed co-expression with *TBL27* in Arabidopsis’s roots, implying that *MYB103* may be associated with *TBL27*. More importantly, increased Al accumulation of cell wall and hemicellulose in *myb103* mutants’ root was ascribed to the changes in the *O*-acetylation level of xyloglucan rather than the xyloglucan content itself. To further understand this MYB103-mediated regulatory pathway in this Al-responsive process, we revealed that MYB103-TBL27 regulatory module affects Al sensitivity through modulating the *O*-acetylation level of cell wall xyloglucan using the approach of biochemistry, genetics and molecular biology comprehensively.

Except for adaptative response in Al toxicity, the cell wall itself or its modification is also crucial for nutritional deficiencies or other heavy metal toxicity. For example, it is interesting that cadmium (Cd) toxicity can be alleviated in *Arabidopsis* by decreasing cell wall polysaccharides (hemicellulose) contents. Therefore, less Cd is stored in the root cell wall, indicating that the cell wall are indeed involved in root Cd exclusion.^13,14^ Under Fe deficiency, it is demonstrated that phenolic compounds are secreted to release cell wall-retained Fe in red clover; thus, cell wall Fe is reutilized and Fe homeostasis is maintained.^15^ Next, it raises the question: which cell wall composition or modification contributes to this Fe remobilization? Lei et al. illustrates that hemicellulose, especially for hemicellulose 1 fraction, is associated with root cell wall Fe reutilization.^16^ Pectin, a significant component of cell walls that includes galactans, homogalacturonan (HGA), arabinans, rhamnogalacturonans I (RG I), RG II and other polysaccharides, can help the reutilization of the phosphorus (P) from the cell wall-deposited P.^17^ Recently, it is also discovered that the methylesterification degree of pectin, controlled by pectin methylesterases, plays a vital role in enhancing root cell wall P remobilization in P-deficient rice.^18^ However, to our knowledge, the molecular mechanisms of how cell wall compositions or modifications are involved in these abiotic stresses are still elusive, which needs further investigation.

Soils with heavy metal contamination or nutrients deficiency are widely distributed in the world, while it is difficult and costly to repair. Therefore, enhancing plants’ resistance to these environmental stresses is the first choice to reduce the metal toxicity or increase nutrients uptake in soils. Compared with other mechanisms, the effect of the cell wall in plant tolerance to these abiotic stresses is rarely studied. Although some achievements have been made in this area, more researchers need to be involved in this field to systematically explore the molecular physiological mechanisms of cell wall’s reaction to above stresses and to solve the metal toxicity/nutrients deficient problem in soils. In this paper, we mainly focus on the changes in the cell wall’s binding properties under Al stress and point out the underlying mechanisms in regulating cell wall composition or cell wall modification to respond to Al toxicity and this provides a good start for further exploring Al detoxification mechanisms. Furthermore, we also briefly introduce the research progress of different cell wall compositions or modifications in other abiotic stresses such as Fe deficiency (Figure 1). However, due to the limitation in mutants related to pectin and hemicellulose modifications, the genetic basis of cell wall modifications in adaption to environmental stress is still unknown. Therefore, in the future, the contribution rate of various polysaccharides of pectin/hemicellulose in the cell wall should be clarified, and systematic research should be carried out on major grain crops in order to obtain a new way to effectively solve these abiotic stresses in crops from the perspective of modifying cell wall compositions.

**Figure 1.**
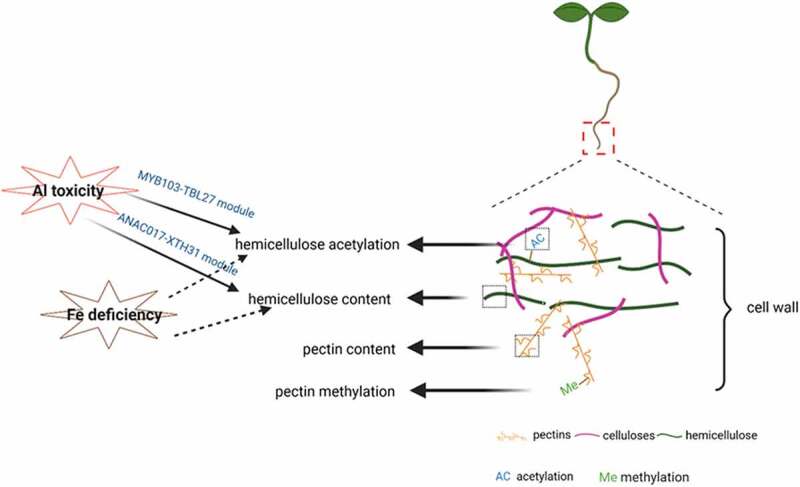
Changes of cell wall characteristics under the abiotic stress.
